# Identification of miRNA Reference Genes in Extracellular Vesicles from Adipose Derived Mesenchymal Stem Cells for Studying Osteoarthritis

**DOI:** 10.3390/ijms20051108

**Published:** 2019-03-05

**Authors:** Enrico Ragni, Carlotta Perucca Orfei, Paola De Luca, Alessandra Colombini, Marco Viganò, Gaia Lugano, Valentina Bollati, Laura de Girolamo

**Affiliations:** 1 IRCCS Istituto Ortopedico Galeazzi, Laboratorio di Biotecnologie Applicate all’Ortopedia, 20161 Milan, Italy; carlotta.perucca@grupposandonato.it (C.P.O.); deluca.paola@grupposandonato.it (P.D.L.); alessandra.colombini@grupposandonato.it (A.C.); marco.vigano@grupposandonato.it (M.V.); gaia.lugano@grupposandonato.it (G.L.); laura.degirolamo@grupposandonato.it (L.d.G.); 2 Università degli Studi di Milano, EPIGET—Epidemiology, Epigenetics and Toxicology Lab, Department of Clinical Sciences and Community Health, 20122 Milan, Italy; valentina.bollati@unimi.it

**Keywords:** adipose-derived mesenchymal stem cells, osteoarthritis, extracellular vesicles, miRNA, reference genes

## Abstract

Osteoarthritis (OA) leads to chronic pain and disability, and traditional conservative treatments are not effective in the long term. The intra-articular injection of mesenchymal stem cells (MSCs) is considered a novel therapy for OA whose efficacy mainly relies on the adaptive release of paracrine molecules which are either soluble or extracellular vesicles (EVs) embedded. The correct quantification of EV-miRNAs using reliable reference genes (RGs) is a crucial step in optimizing this future therapeutic cell-free approach. The purpose of this study is to rate the stabilities of literature-selected proposed RGs for EV-miRNAs in adipose derived-MSCs (ASCs). EVs were isolated by ultracentrifugation from ASCs cultured with or without inflammatory priming mimicking OA synovial fluid condition. Expression of putative RGs (let-7a-5p, miR-16-5p, miR-23a-3p, miR-26a-5p, miR-101-3p, miR-103a-3p, miR-221-3p, miR-423-5p, miR-425-5p, U6 snRNA) was scored by using the algorithms geNorm, NormFinder, BestKeeper and ΔCt method. miR-16a-5p/miR-23a-3p yielded the most stable RGs, whereas let-7a-5p/miR-425-5p performed poorly. Outcomes were validated by qRT-PCR on miR-146a-5p, reported to be ASC-EVs enriched and involved in OA. Incorrect RG selection affected the evaluation of miR-146a-5p abundance and modulation by inflammation, with both values resulting strongly donor-dependent. Our findings demonstrated that an integrated approach of multiple algorithms is necessary to identify reliable, stable RGs for ASC-EVs miRNAs evaluation. A correct approach would increase the accuracy of embedded molecule assessments aimed to develop therapeutic strategies for the treatment of OA based on EVs.

## 1. Introduction

Osteoarthritis (OA) is the 11th cause of disability in the world [1]. In 2012, the proportion of population aged ≥45 with OA was 27%, with the most common locations being knee (14%), hip (6%) and hand (3%). Notably, by 2032 this number is expected to increase to 30% [2], representing a significant economic burden for society and patients [3]. Both loss of cartilage volume and cartilage lesions associated with inflammation of the articular structures are distinctive traits of the pathology of OA joint [4]. Currently, the primary strategy for mild to moderate OA treatment is to reduce pain and improve function and quality of life, mainly using medications such as nonsteroidal anti-inflammatory drugs, opioids and corticosteroids [5]. Surgical interventions, including joint debridement, are recommended when the progression of OA has resulted in severe damage to the joint, with joint replacement to be considered the final option [6]. However, none of the prevailing therapies have been shown to protect articular cartilage or prevent OA evolution [7], with an unmet medical demand for treatments that can halt the progression of the disease providing long-term relief from the symptoms.

Mesenchymal stem cells (MSCs) are a good candidate to meet the challenge in treating OA. MSCs are ubiquitous throughout the musculoskeletal system given their perivascular localization [8,9]. The most frequently isolated MSCs are from bone marrow (BMSCs) [10] and adipose tissue (ASCs), with the last being the favorite choice due to ease of tissue harvest and high abundance [11]. Clinical trials involving MSCs in OA patients have recently begun, contributing to a better understanding of cell-based therapies for degenerative joint diseases [12,13]. Nevertheless, although they have shown a positive effect on OA patients, exploring the mechanisms underlying symptom-modifying MSCs treatment is crucial for developing standardized protocols.

In this context, the evidence supporting the paracrine actions of MSCs is continuously growing [14,15]. Indeed, MSCs release adaptively a wide number of factors (cytokines, chemokines, immunomodulatory and growth factors), with additional paracrine molecules, such as cytokines and growth factors, signaling lipids, mRNAs, and regulatory miRNAs, encapsulated in extracellular vesicles (EVs) [14,16]. In the OA field, pre-clinical studies demonstrated the efficacy of MSC-EVs in different settings, including collagenase-induced OA [17,18], osteochondral defects [19], destabilized medial meniscus- induced OA [20], and glucocorticoid-induced osteonecrosis of the femoral head [21]. Similarly, MSC-EVs showed an anti-inflammatory effect in an antigen-induced swine model of synovitis [22].

To ensure the most effective MSC-EVs therapeutic potential and develop standardized and reproducible protocols, cargo composition and molecular mechanisms have to be dissected to unravel the array of conveyed active molecules and potentially tune their concentration. Recent studies have assigned a major role to MSC-EVs miRNAs in modulating target cell function [16]. Thus, characterizing and further enriching EVs with genetic materials such as therapeutically-functional miRNAs may be of particular relevance. From this perspective, a major pitfall is the reliable quantification and comparison of EVs associated miRNAs between samples or donors due to the lack of adequate miRNA-reference genes (RGs). In fact, in MSC-EVs, a consensus has not yet been provided for either cellular mRNAs or miRNAs [23,24,25]. Therefore, the purpose of this study was to identify the most stable miRNA RGs in EVs isolated from adipose-derived MSCs cultured with or without inflammatory priming mimicking OA synovial fluid, a condition that ASCs encounters when injected in the joint. The identification of reliable RGs in normal and pro-inflammatory conditions would be crucial for both basic research and for clinical approaches where the accuracy of EV content assessment is mandatory to produce the most effective and standardized product.

## 2. Results

### 2.1. ASCs and EVs Characterization

**Figure 1:**
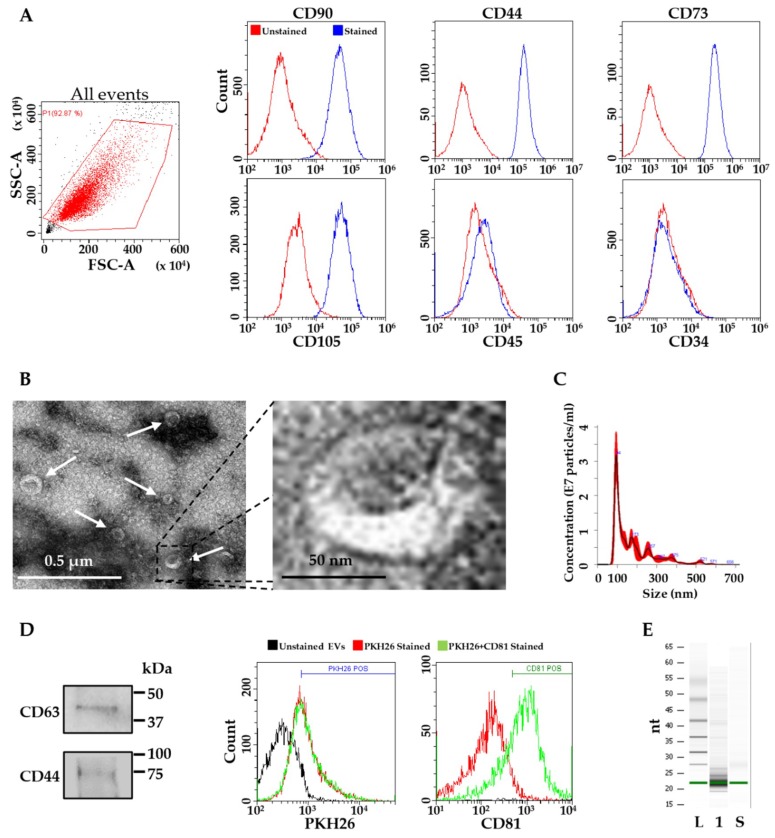
Characterization of ASCs and ASC-EVs. (**A**) Flow cytometry analysis of mesenchymal positive (CD90, CD44, CD73 and CD105) / negative (CD45) and hematopoietic (CD34) stem cell markers confirming ASC identity. Representative plots of ASC1 are shown; (**B**) Electron microscopy of ASC-EVs (white arrows in negative-stain images) with 10× magnification of a representative EV field; (**C**) Representative nanotracking analysis of EVs; (**D**) Western blot showing the presence of EV (CD63) and MSC (CD44) markers on ASC-EVs and flow cytometry scoring CD81 EV marker positivity on PKH26-labeled ASC-EVs; (**E**) Representative Bioanalyzer profiles of ASC-EVs, the small RNAs corresponding to 20–25 nt were dominant. L stands for Ladder, 1 for RNA extracted from ASC1-EVs and S for synthetic 22 nt small RNA.

The stem cell identity of isolated ASCs was assessed by flow cytometry. The analysis confirmed a high expression of typical MSC cell-surface antigens, including CD44, CD73, CD90 and CD105, negativity for blood line marker CD45 and absence of hematopoietic stem cell marker CD34 (Figure 1A). EVs isolated from ASCs were analyzed by transmission electron microscopy and Nanoparticle tracking analysis (NTA). ASC-EVs exhibited the characteristic cup-shape morphology (Figure 1B), and were within the normal vesicle size range (50–400 nm in diameter), with enrichment in the small ones (mode size 100 ± 8 nm) (Figure 1C). ASC-EVs expressed both CD63 and CD 81, consistent with previously reported characteristics of EVs (Figure 1D). CD44, a MSC-EVs defining marker, was also detected (Figure 1D).

Total RNA derived from ASC-EVs was analyzed using Agilent Bioanalyzer small RNA chips (Figure 1E). The majority of RNA content was between 20 and 30 nt, indicating that ASC-EVs are enriched in short RNAs such as miRNAs, containing about 21–25 nucleotides.

### 2.2. Expression of Candidate Reference Genes

**Figure 2:**
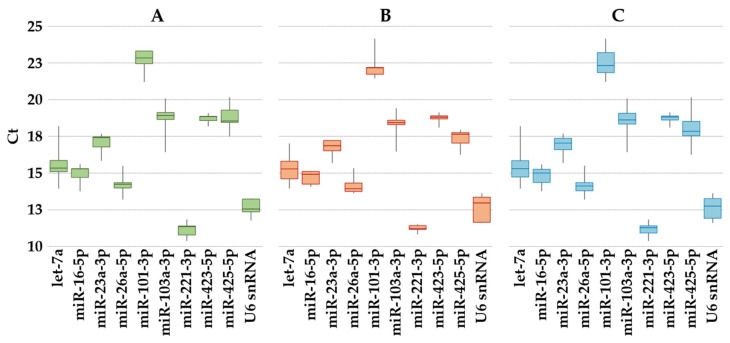
Expression of candidate reference genes in ASC-EVs. The box plot graphs of the Ct values for each reference gene illustrate the interquartile range (box) and median. The whisker plot depicts the range of the values. (**A**) ASC without priming; (**B**) ASC after inflammatory stimuli; (**C**) All the studied samples.

The expression of the 10 selected reference genes (Table 1) was assayed in EVs samples from the three groups (ASC without priming; ASC with inflammatory stimuli; all the studied samples). miR-101-3p had the lowest expression, whereas miR-221-3p had the highest under all conditions (Figure 2A–C). Moreover, none of them resides within the same gene cluster, which reduces the likelihood of including coregulated miRNAs in the analysis [26].

### 2.3. Reference Genes Expression Stability Analysis

In order to rank the stability of the selected RGs, four algorithms were used (NormFinder, geNorm, BestKeeper, and the comparative ΔCt method) (Table 2). In ASCs samples, NormFinder analysis identified miR-101-3p as the most stably expressed RG, with a stability value of 0.014, followed by miR-23a-3p (0.06) and miR-16-5p (0.73). Under inflammation, the three most stable miRNA were miR-23a-3p, miR-16-5p and miR-423-5p (0.099, 0.144 and 0.158, respectively). Finally, considering the whole ASCs with and without priming, miR-23a-3p again showed the highest stability (0.057), followed by miR-16-5p (0.087) and miR-101-3p (0.143).

GeNorm identified miR-23a-3p/miR-16-5p (0.044) and miR-101-3p (0.165) as the most stable candidates. In ASCs treated with inflammatory cytokines, miR-221-3p/miR-423-5p (0.209) was the most stably expressed, followed by miR-16-5p (0.0289). Combining all samples, miR-16-5p/miR-23a-3p (0.212) were found as less variable, then miR-423-5p (0.345).

Using BestKeeper, in ASCs miR-123-5p (0.26), miR-221-3p (0.45) and U6 (0.49) showed the lowest SD variation. In OA conditions, miR-221-3p (0.19) was the most reliable, followed by miR-423-5p (0.26) and miR-16-5p (0.43). When both the conditions were analyzed together, the BestKeeper ranking was miR-423-5p (0.26), miR-221-3p (0.32) and miR-16-5p (0.49).

The comparative Δ*C*t method results are presented. The outcomes of the ΔCt method were similar as those of the geNorm analysis, with the three most stable RGs being: i) miR-23a-3p (0.526), miR-16-5p (0.530) and miR-101-3p (0.563) for ASCs; miR-23a-3p (0.645), miR-16-5p (0.647) and miR-423-5p (0.652) under inflammatory condition; and miR-16-5p (0.616), miR-23a-3p (0.618) and miR-423-5p (0.687) when all the samples were assayed together.

Since the various software programs generated different results, integration and normalization of the data was mandatory. Geomean of each candidate weight across the four algorithms was calculated to re-rank RGs accordingly. The gene with the lowest value was considered to be the most stable. In ASCs, the three most stable RGs were miR-23a-3p, miR-16-5p and miR-101-3p, whereas those which scored the worst were let-7a-5p, miR-103a-3p and miR-425-5p. Under inflammatory condition, miR-23a-3p, miR-221-3p and miR-423-5p were the most stable, with the least stable being U6 snRNA, miR-425-5p and let-7a-5p. Considering all samples, miR-16-5p, miR-23a-3p and miR-423-5p resulted the most reliable whereas miR-425-5p, let-7a-5p and U6 snRNA should be avoided.

### 2.4. Impact of RGs Choice on the Expression Levels of Target Genes

**Figure 3:**
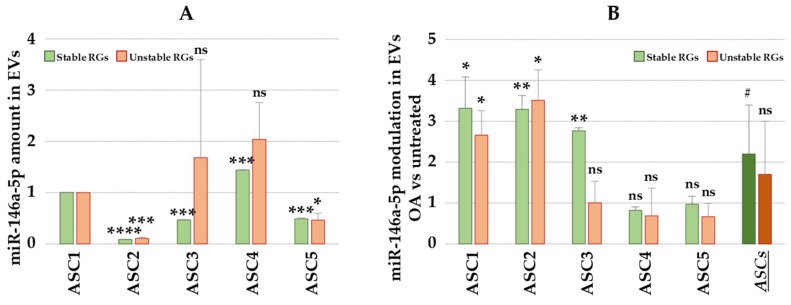
Effects of different reference genes on the normalization of miR-146a-5p expression. Best RGs represents miR16-5p and miR-23a-3p whereas Worst RGs stands for the combination of let-7a-5p and miR-425-5p. (**A**) miR-146a-5p expression in the five ASC-EVs. ASC1-EVs used as reference for statistical significance (ns = not statistically significant; * *p*-value ≤ 0.05; *** *p*-value ≤ 0.001; **** *p*-value ≤ 0.0001). (**B**) Differential expression of miR-146a-5p in OA inflamed *vs* resting ASC-EVs. ASCs stands for all five samples grouped. Untreated cells used as reference for statistical significance (ns = not statistically significant; ^#^ *p*-value ≤ 0.1; * *p*-value ≤ 0.05; ** *p*-value ≤ 0.01).

qRT-PCR assays on miR-146a-5p were performed in order to further evaluate the reliability of the selected candidate RGs in the paired sample set (OA vs untreated). miR-146a-5p expression level data were normalized using the combination of the two most stable (miR16-5p and miR-23a-3p) and the two least stable (let-7a-5p and miR-425-5p) RGs. Interestingly, in the five ASC-EVs assessed in the study, miR-146a-5p showed different basal levels (Figure 3A), with Ct values between 19 for ASC4-EVs and 23 for ASC2-EVs. Notably, in ASC3-EVs, the normalization approach strongly influenced the evaluation leading to the misleading conclusion: indeed, using unreliable RGs such as let-7a-5p (10 times higher than real value), the amount of miR-146a-5p appeared to be 3.6 times higher. Moreover, in ASC4-EVs, although unstable RGs gave a result similar to the one obtained with optimal RGs, this did not make it possible to observe any statistical significance. Next, the effect of OA-like cytokines on normalization of miR-146a-5p expression was assessed (Figure 3B). When using the appropriate RGs, ASC1-EVs, ASC2-EVs and ASC3-EVs showed significant increase of EV-associated miR-146a-5p (*p*-value ≤ 0.05), whereas ASC4-EVs and ASC5-EVs did not show any modulation. Again, the incorrect selection of RGs led to misleading scores in ASC3-EVs. Finally, since in a previous publication on EVs from heavily inflamed (20 ng/mL IFNγ/TNFα) ASCs miR-146a-5p resulted 3 times upregulated [36], the analyzed samples were grouped. Only the most reliable RGs allowed us to assess the overall increase (R ≥ 2, *p*-value ≤ 0.1), albeit while keeping in mind the differences between ASC-EVs samples.

## 3. Discussion

In this work, a rigorous method to identify and validate reference genes in ASC-EVs has been proposed. miR-16a-5p/miR-23a-3p yielded the most stable RGs, whereas let-7a-5p/miR-425-5p performed poorly. Incorrect RG selection affected the reliable evaluation of potentially therapeutic miRNAs, as OA-related miR-146a-5p.

ASC-EVs are a therapeutically suitable tool in degenerative diseases, such as joint degeneration and osteoarthritis, both enhancing the therapeutic effects of ASCs transplantation and as an off-the-shelf cell-free clinical option [37]. The exponential increase of basic and translational studies on EVs has highlighted the need for reliable RGs in order to unravel their molecular mechanisms, modulations in cargo components due to different conditions of secreting cells culturing, and differences between EV sources in terms of tissue origin. In this view, ASC-EVs were used as a model for miRNA RGs identification strategy, given the paucity of available data about EVs reference genes, through a qRT-PCR approach.

To date, independently of the nucleic acid source (cells or EVs), few alternative normalization approaches for miRNA qRT-PCR validation are available. The strategy which is capable of obtaining the most stable RGs within independent samples is the normalization by global mean miRNA expression [26]. However, the major limit of this method is the remarkable amount of required RNA, that must be enough to score the entire miRNome, along with high costs and long time required to interpret the results. The second approach relies on the addition of a spike-in control in equal amount of samples, like the synthesized miRNA from Arabidopsis thaliana ath-miR-159a we supplemented in a similar ASC-EVs amount before RNA extraction. Although it may be very useful to estimate the influence of the technical workflow, the endogenous state of the overall miRNA expression cannot be considered to be reliable [38,39,40]. To overcome these limitations, the endogenous control method, which relies on the relative expression of the target gene using stable and abundant endogenous candidates, is preferred [41,42,43]. In this view, U6 snRNA is a common RG for the relative quantification of target miRNAs. U6 snRNA is a nuclear transcript involved in the spliceosome complex of mRNA transcription with no post-transcriptional signaling functions. Although thought to be exclusively expressed in the nuclei, and therefore, not detectable in isolated vesicles, few studies recommend U6 snRNA as a RG for quantification of miRNA in EVs or vesicle-enriched body fluids [44,45]. In our samples, U6 snRNA never performed as the most stable reference gene, being in the last position when ASCs were inflamed, and was among the worst candidates when ASCs in both conditions were assessed together. Similar results were obtained in a recent paper scoring RGs in cardiosphere-derived cell EVs. This may depend on U6 snRNA biogenesis that is mechanistically separated from miRNA biogenesis, which is not processed by the spliceosome but by the Drosha complex [46]. Therefore, a crucial issue appears to be the selection of the reference RNA on the class of RNAs being investigated, suggesting that reference miRNAs would be preferable for miRNAs.

Regardless of the computational approaches, our study showed a superior stability for miR-23a-3p, miR-16-5p and miR-423-5p in all conditions tested, whereas miR-221-3p was stable only in cells in basal conditions. Data integration was necessary, since the different algorithms generated slightly different results depending on the specific computational approaches. In fact, geNorm and BestKeeper are pair-wise based, selecting the most suitable RGs on the variation of expression ratios between the genes across the sample sets, although geNorm includes pairs of co-regulated genes based on their similar expression profiles. Differently, NormFinder and the comparative ΔCt method are designed to eliminate the effects of co-regulation. miR-23a-3p was already reported to be among the most expressed miRNAs in EVs from umbilical cord- [47] and bone marrow-derived MSCs [48], a crucial trait for a RG that, upon further validation, may be used across MSCs from different sources. Interestingly, in OA tissues such as cartilage, a negative-feedback regulatory mechanism involving inflammatory signals was reported to tune the expression of miR-23a-3p [49]. Regarding miR-16-5p, it was reported to be highly expressed in bone marrow-derived MSCs but not among the top 20 in derived EVs [50], making this molecule less likely to be a potential RG across EVs from different MSCs. miR-16-5p was also reported to be a regulator of SMAD3 expression in human chondrocytes possibly contributing to the development of OA [51]. Finally, miR-423-5p was reported as highly and exclusively expressed in bone marrow derived-MSC EVs [35], and therefore, a future candidate to be scored as general MSC-EVs RG. Notably, miR-423-5p was identified as a miRNA normalizer between healthy and OA tissues [52]. Therefore, with future data regarding the expression of these miRNAs in EVs derived from other MSCs, it will be possible to find stable and highly-expressed RGs to predict the potency of vesicles in mesenchymal stem cells from similar or different donor and tissue sources.

With regards to cargo dissection and potency prediction, the effects of the normalization strategies using stable or unstable RGs were analyzed on miR-146a-5p. miR-146a-5p was shown to control both OA-associated pain and homeostasis of the knee joint through a strict balance of the inflammatory response in cartilage and synovium [53]. Furthermore, it has been demonstrated that miR-146a-5p regulates cytokine signaling through a negative feedback loop, suggesting that its administration might be a candidate for new treatments for the early stage of OA [54]. Also, importantly, miR-146a-5p participates in macrophage polarization toward M2 phenotype [55]. Therefore, an accurate quantification of its expression and a reliable evaluation of its modulation may be crucial to add information to the overall ASCs and ASC-EVs therapeutic potential in OA, since miR-146a-5p was found to be abundant in ASC-EVs and upregulated by inflammatory priming [35,36]. Herein presented data showed that miR-146a-5p amount and its increase upon inflammation is strongly donor-depending; this is a crucial point to be considered. In fact, reporting data about regulatory and effector molecules levels and modulation in term of overall values, as previously published for miR-146a-5p for ASC-EVs [35,36], may result in misleading potency predictions, albeit useful ones for a general overview. Therefore, miRNA presence evaluation, together with other small molecules in different MSC types to forecast the most therapeutically-effective choice, will be mandatory through a rigorous dissection of single isolates. Hence, strict validation of RG suitability results is crucial to help in predicting the overall (miRNAs, cytokines, lipids) efficacy of both cells and their derived EVs at both cell type and donor levels, in the setting of future off-the-shelf clinical products.

In conclusion, further studies are needed to investigate the utility of miRNA RGs with the growing list of other EVs isolated from different MSC types. From a basic science and discovery perspective, dissecting EV molecular cargo will greatly increase the translational potential of extracellular vesicles. This will be crucial to identify both the most suitable MSC-EV for the target pathology and, even more importantly, to score potency of each EV isolate. Moreover, to foster a quick and effective translational development, the selection of appropriate RGs will further strengthen independent gene expression studies for production process development, scale-up and quality control.

## 4. Materials and Methods

### 4.1. Ethics Statement

This work was performed at IRCCS Istituto Ortopedico Galeazzi with Institutional Review Board approval and specimens were collected under patient informed consent (M-SPER-015—Ver. 2—04.11.2016), and following the 1964 Helsinki declaration and its later amendments or comparable ethical standards.

### 4.2. ASCs Isolation and Expansion

Human adipose tissue samples were obtained from waste material of five female donors (range 61-44 years old) who had undergone elective plastic surgery. 0.075% *w*/*v* type I collagenase (Worthington Biochemical Co, Lakewood, NJ, USA) was used to digest adipose tissue (37 °C, 30 min). After digestion, samples were filtered through a cell strainer and centrifuged (1000× *g*, 5 min) [56]. Cells in the pellet were seeded at 5 × 10^3^ cells/cm^2^ in DMEM supplemented with 10% FBS (GE Healthcare, Piscataway, NJ, USA), L-glutamine and pen/strepto (Life Technology, Carlsbad, CA, USA). Culture were kept at 37 °C, 5% CO_2_ and 95% humidity. To mimic inflammatory environment, ASCs were treated with cytokines resembling those quantified in OA synovial fluid (40 pg/mL IFNγ, 10 pg/mL Il-1β and 5 pg/mL TNFα) [57]. Cells were characterized by flow cytometry using positive or negative MSC markers (CD44, CD73, CD90 and CD105 or CD45; Miltenyi Biotec, Bergisch Gladbach, Germany) and hematopoietic negative marker (CD34) [58,59] as described previously with a CytoFLEX flow cytometer (Beckman Coulter, Fullerton, CA, USA) collecting a minimum of 30,000 events [60]. Cells were used for the experiments between passage 3 and 5.

### 4.3. ASC-EVs Isolation and Characterization

At 90% cell confluence (approximately 10,000 cells/cm^2^, corresponding to 1.5 population doublings with respect to initial sowing of 3000 cells/cm^2^), T175 culture flasks were washed three times with PBS and 12 mL DMEM without FBS added. For samples under OA resembling inflammation, 40 pg/mL IFNγ, 10 pg/mL Il-1β and 5 pg/mL TNFα (PeproTech, Rocky Hills, NJ, USA) were added to DMEM during vesicle release. After 48 h, conditioned medium was collected and subjected to differential centrifugation as already described [60] with few modifications. Briefly, debris were removed by centrifugation at 376× *g* for 15 min. The supernatant was further centrifuged at 1000× *g* for 15 min, then 2000× *g* for 15 min and finally twice at 4000× *g* for 15 min with all steps performed at 4 °C. EVs in 10.5 mL were recovered by ultracentrifugation at 100,000× *g* for 9 h at 4 °C in a 70Ti rotor (Beckman).

Western blotting: EVs were dissolved in Laemmli Buffer (Bio-Rad, Hercules, CA, USA) at 37 °C for 30 min. 5 μg of protein extracts were separated on 4–20% Mini-PROTEAN TGX™ Precast Protein Gels (Bio-Rad) before being blotted onto PVDF (Bio-Rad). Membranes were blocked at 4 °C with TBS-T—5% milk overnight and subsequently CD63 (Miltenyi Biotec) [61] or CD44 (Immunostep, Salamanca, Spain) [62] mouse anti-human primary antibodies were used for immunodecoration for 2 h at RT. Anti-mouse IgG HRP-linked secondary antibody were used to visualize bound proteins by ChemiDoc™ Imaging Systems (Bio-Rad).

Flow cytometry: After ultracentrifugation at 190,000× *g* for 3 h at 4 °C, EVs pellet was suspended in 1 mL of Diluent C and 6 μL PKH26 (Sigma-Aldrich, St. Louis, MO, USA) added. Incubation was performed for 5 min at RT and dye quenched by adding 2 mL 10% BSA. 1.5 mL of a 0.971 M sucrose solution was added into the bottom of the tube and solution centrifuged at 190,000× *g* at 4 °C for 2 h. Pellet was suspended in PBS and stained with anti CD81 (Becton Dickinson, NJ, USA) for 30 min at 4 °C in the dark. Collection was performed with a CytoFLEX flow cytometer collecting a minimum of 30,000 events.

Transmission electron microscopy: Formvar carbon-coated grids were used to absorb 5 µL of purified EVs for 10 min. The drops were blotted with filter paper. A 2% uranyl acetate aqueous suspension was used for 10 min for negative staining. The excess of uranyl was removed by filter paper by touching the grid. The grid was dried at room temperature. Samples were examined with a TALOS L120C transmission electron microscope (Thermo Fisher Scientific, Waltham, MA, USA) at 120 kV.

Nanoparticle tracking analysis (NTA): Nanosight LM10-HS system (NanoSight Ltd., Amesbury, UK) was used to visualize EVs by laser light. Three recordings of 30 s were performed for each EV sample. NTA software analyzed collected data, providing high-resolution particle size distribution profiles and concentration measurements.

### 4.4. Selection of Candidate Reference Genes

According to previously published papers scoring EV-miRNA RGs in a variety of tissues or cell types [27,28,29,30,31,32,33,34,63], nine miRNAs (let-7a-5p, miR-16-5p, miR-23a-3p, miR-26a-5p, miR-101-3p, miR-103a-3p, miR-221-3p, miR-423-5p and miR-425-5p) and one small RNA (U6 snRNA) were selected to be evaluated as possible candidates (Table 1).

### 4.5. Total RNA Isolation and miRNA Profiling

Total RNA was isolated from similar EV numbers (10 ± 3 E9) using the miRNeasy Kit and RNeasy Cleanup Kit (Qiagen, Hilden, Germany), following the manufacturer’s instruction. To monitor the RNA recovery procedure, before RNA extraction, samples were spiked-in with exogenous ath-miR-159a (30 pg), an *Arabidopsis thaliana* synthetic miRNA whose specific primers are provided in the RT and PreAmp primer pools (Life Technologies, Foster City, CA, USA). miRNA cDNA samples were prepared by standard reverse transcription (RT) and preamplification procedures, as previously reported [64]. Preamplified samples were stored at −20 °C until expression analysis with the OpenArray system (Life Technologies) performed into 384-well OpenArray plates, according to manufacturer’s instructions. For each amplification curve, an AmpScore value was obtained. miRNAs with AmpScore < 1.1 or missing and Ct values > 27 were considered unamplified. The following assays (Life Technologies) were considered: hsa-miR-23a-3p 000399; hsa-miR-221-3p 000524; hsa-miR-423-5p 002340; hsa-miR-16-5p 000391; hsa-miR-26a-5p 000405; hsa-miR-103a-3p 000439; hsa-miR-101-3p 002253; hsa-let-7a-5p 000377; hsa-miR-425-5p 001516; U6 snRNA 001973; hsa-miR-146a-5p 000468; ath-miR159a 000338.

### 4.6. Data Analysis

Before RG analysis, the efficiency of the whole process, from RNA isolation to amplification, was evaluated by ath-miR159 Ct values; this was shown to be extremely reproducible (18.33 ± 0.48) across all samples. Therefore, ath-miR159 Ct values were used for a first technical normalization of the other miRNA Ct values before data analysis. The expression stability of the ten candidate RGs was calculated by four algorithms: geNorm [65], NormFinder [66], BestKeeper [67] and the comparative ΔCt method [68]. These four approaches rated the expression stability according to different variables. Linear scale quantitative data is the basis for Normfinder analyses of RG expression stability. This allowed us to identify a stability value for each investigated candidate. Higher stability is indicated with a lower stability value. GeNorm provides an M-value based on the average pairwise expression ratio. A lower M-value indicates more stable expression, and RGs with M ≤ 1.5 are considered to be stably expressed. BestKeeper identifies RG stability according to SD, with a higher SD indicating a less stably-expressed candidate. The ranking of the RGs according to their stability was generated by each approach, and a series of continuous integers starting from 1 was assigned to each RG. The geometric mean (geomean) of each RG weight across the four programs was subsequently determined, after which the RGs were re-ranked accordingly. The RG with the lowest geomean was considered to be the most stable.

### 4.7. Statistical Analysis

Statistical analyses were performed using GraphPad Prism Software version 5 (GraphPad, San Diego, CA, USA). Data were scored for normality by Kolmogorov-Smirnov test. The comparison between the groups was performed by using unpaired Student t-test. Significance level was set at *p*-value ≤ 0.05.

## Figures and Tables

**Table 1:** Candidate reference genes and target gene primer sequences.

Accession Number	Gene Name	Target Sequence (5′–3′)	Reference
Candidate reference genes			
MIMAT0000062	let-7a-5p	UGAGGUAGUAGGUUGUAUAGUU	[27,28,29]
MIMAT0000069	miR-16-5p	UAGCAGCACGUAAAUAUUGGCG	[30,31]
MIMAT0000078	miR-23a-3p	AUCACAUUGCCAGGGAUUUCC	[32]
MIMAT0000082	miR-26a-5p	UUCAAGUAAUCCAGGAUAGGCU	[28,32]
MIMAT0000099	miR-101-3p	UACAGUACUGUGAUAACUGAA	[32]
MIMAT0000101	miR-103a-3p	AGCAGCAUUGUACAGGGCUAUGA	[27]
MIMAT0000278	miR-221-3p	AGCUACAUUGUCUGCUGGGUUUC	[27,28]
MIMAT0004748	miR-423-5p	UGAGGGGCAGAGAGCGAGACUUU	[33]
MIMAT0003393	miR-425-5p	AAUGACACGAUCACUCCCGUUGA	[33]
NR_004394.1	U6 snRNA	GUGCUCGCUUCGGCAGCACAUAUACUAAAAU UGGAACGATACAGAGAAGAUUAGCAUGGCCC CUGCGCAAGGAUGACACGCAAAUUCGUGAAG CGUUCCAUAUUUU	[34]
miRNA target			
MIMAT0000449	miR-146a-5p	UGAGAACUGAAUUCCAUGGGUU	[35]

**Table 2:** Expression levels of candidate reference genes

Gene Name	GeNorm *M*-value	NormFinder Stability	BestKeeper SD ± CP	ΔCt Mean	Geomean	Ranking Order
**ASCs**						
miR-23a-3p	0.044 (1)	0.060 (2)	0.579 (6)	0.526 (1)	1.86	1
miR-16-5p	0.044 (1)	0.076 (3)	0.554 (5)	0.530 (2)	2.34	2
miR-101-3p	0.165 (3)	0.014 (1)	0.641 (7)	0.563 (3)	2.82	3
miR-423-5p	0.369 (5)	0.327 (6)	0.232 (1)	0.672 (5)	3.50	4
U6 snRNA	0.315 (4)	0.229 (4)	0.490 (3)	0.628 (4)	3.72	5
miR-26a-5p	0.427 (6)	0.260 (5)	0.533 (4)	0.679 (6)	5.18	6
miR-221-3p	0.481 (7)	0.478 (8)	0.451 (2)	0.810 (7)	5.29	7
miR-425-5p	0.538 (8)	0.505 (9)	0.747 (8)	0.850 (8)	8.24	8
miR-103a-3p	0.612 (9)	0.474 (7)	0.885 (9)	0.868 (9)	8.45	9
let-7a-5p	0.735 (10)	0.812 (10)	1.071 (10)	1.227 (10)	10.00	10
**OA-Like Inflamed-ASCs**						
miR-23a-3p	0.338 (4)	0.099 (1)	0.477 (4)	0.645 (1)	2.00	1
miR-221-3p	0.209 (1)	0.257 (4)	0.187 (1)	0.695 (4)	2.00	2
miR-423-5p	0.209 (1)	0.158 (3)	0.263 (2)	0.652 (3)	2.06	3
miR-16-5p	0.289 (3)	0.144 (2)	0.428 (3)	0.647 (2)	2.45	4
miR-26a-5p	0.442 (5)	0.318 (5)	0.506 (5)	0.747 85)	5.00	5
miR-103a-3p	0.551 (6)	0.423 (6)	0.709 (7)	0.852 (6)	6.24	6
miR-101-3p	0.630 (7)	0.567 (7)	0.722 (8)	0.961 (7)	7.24	7
let-7a-5p	0.678 (8)	0.575 (8)	0.862 (10)	0.974 (8)	8.46	8
miR-425-5p	0.837 (10)	0.732 (10)	0.545 (6)	1.150 (10)	8.80	9
U6 snRNA	0.759 (9)	0.606 (9)	0.808 (9)	1.044 (9)	9.00	10
**ALL ASCs**						
miR-16-5p	0.212 (1)	0.087 (2)	0.491 (3)	0.616 (1)	1.57	1
miR-23a-3p	0.212 (1)	0.057 (1)	0.528 (5)	0.618 (2)	1.78	2
miR-423-5p	0.345 (3)	0.190 (5)	0.262 (1)	0.687 (3)	2.59	3
miR-221-3p	0.384 (4)	0.230 (7)	0.316 (2)	0.781 (5)	4.09	4
miR-26a-5p	0.474 (5)	0.186 (4)	0.525 (4)	0.717 (4)	4.23	5
miR-101-3p	0.532 (6)	0.143 (3)	0.739 (7)	0.788 (6)	5.24	6
miR-103a-3p	0.644 (8)	0.229 (6)	0.826 (8)	0.845 (7)	7.20	7
U6 snRNA	0.590 (7)	0.242 (8)	0.650 (6)	0.861 (8)	7.20	8
let-7a-5p	0.723 (9)	0.328 (9)	0.966 (10)	1.070 (9)	9.24	9
miR-425-5p	0.818 (10)	0.350 (10)	0.841 (9)	1.198 (10)	9.74	10

miRNAs are ranked according to gene stability as determined by geomean. The numbers in brackets represent the ranking values, regarded as a recommended final ranking.

## Abbreviations

MSCs	Mesenchymal stem cells
ASCs	Adipose derived-MSCs
EVs	Extracellular vesicles
miRNA	microRNA
qRT-PCR	Quantitative-real time polymerase chain reaction
RGs	Reference genes
OA	Osteoarthritis
